# To Compare the Intravenous Bolus Dose of Propofol With an Equipotent Dose of Intravenous Thiopentone for the Facilitation of Laryngeal Mask Airway Insertion

**DOI:** 10.7759/cureus.31917

**Published:** 2022-11-26

**Authors:** Dilip K Saloi, Prabal Bharali, Indukalpa Das, Jagadish Basumatary, Putul Mahanta

**Affiliations:** 1 Anesthesiology, Tezpur Medical College and Hospital, Tezpur, IND; 2 Anesthesiology, Gauhati Medical College and Hospital, Guwahati, IND; 3 Forensic Medicine and Toxicology, Assam Medical College and Hospital, Dibrugarh, IND

**Keywords:** general anesthesia, minor surgery, thiopentone, propofol, laryngeal mask airway (lma)

## Abstract

Objectives

Insertion of laryngeal mask airway has been facilitated by using a variety of induction agents and their combinations with minimal side effects. The current prospective study is a randomized, double-blind study conducted using induction agents, namely, propofol and its equipotent dose of thiopentone, for laryngeal mask airway insertion, and to compare their side effects in patients undergoing minor surgeries requiring general anaesthesia.

Methods

This prospective study was carried out at the Anaesthesiology and Critical Care Department of Gauhati Medical College and Hospital (GMCH), Assam, India. The randomized, double-blinded study comprised 80 patients aged 18 to 60 years undergoing minor surgeries (≤45 minutes) under general anaesthesia fitting into the American Society of Anesthesiologists (ASA) physical status I and II and Mallampati score (MPS) 1 and 2. The participants were randomly divided into two groups in a 1:1 ratio. Group A (n = 40) received propofol (2.5 mg/kg), while group B (n = 40) received thiopentone (5 mg/kg) injections for induction of anaesthesia. Pre-medication with midazolam (0.04 mg/kg) injection and fentanyl (1.5 mcg/kg) injection was provided to patients in both groups. Post-laryngeal mask airway insertion, parameters like conditions for insertion, time taken for laryngeal mask airway insertion, overall response, and haemodynamic parameters were recorded. The data analysis was executed using equivalence tests considering a two-sided p < 0.05 as significant.

Results

Group A had a higher and statistically significant ease of insertion (p = 0.029). The mean insertion time was notably different between the two groups (p < 0.001). The difference in the overall response to insertion showed no statistical significance in the two groups. Statistically, a significant difference was found in falls in heart rate and various blood pressure levels between the groups (p < 0.001).

Conclusion

Propofol at a rate of 2.5 mg/kg was found to be superior to thiopentone at a rate of 5 mg/kg as far as suppression of upper airway reflexes in laryngeal mask airway insertion.

## Introduction

The most effective and safest technique to secure the airway, administer anaesthetic gases, and prevent aspiration is through endotracheal intubation. However, haemodynamic alterations and sympathoadrenal reactions are typically seen during laryngoscopy and endotracheal intubation [1]. The laryngeal mask airway (LMA) was developed to prevent such unfavourable responses by providing some benefits of endotracheal intubation without the fundamental drawback of visualization and separating the cords by force [2]. The upper airway reflexes must be properly obtunded before inserting the LMA to avoid unfavourable patient reactions such as coughing, gagging, and laryngospasm [3].

Various induction agents and combinations have enabled LMA insertion with the fewest side effects. However, each of these methods possesses some shortcomings, and none of them has so far developed into a widely accepted technique [4]. Although propofol is the most frequently used agent to facilitate the placement of an LMA, this drug is expensive and painful on injection. It causes dose-dependent depression of ventilation and a fall in arterial blood pressure [5]. It would be beneficial to find a less-priced induction technology that is just as effective as propofol [6]. Thiopentone, on the contrary, may not depress airway reflex adequately as much as propofol, resulting in gagging, coughing, head and limb movement, and laryngospasm, which are undesirable for LMA insertion. However, it does not create significant bradycardia or hypotension. Several co-induction agents are introduced in the study to make it an adequate substitute for LMA insertion [7]. The concept of co-induction in anaesthesia has come forward by administering small doses of sedative or other anaesthetic agents to decrease the dose requirement of the induction agent to improve the quality of anaesthesia with improvement in haemodynamic stability and with fewer side effects.

Literature comparing the intravenous (IV) bolus dose of propofol and thiopentone for LMA insertion is limited. Though some research exists comparing these two induction agents for LMA insertion, research is limited to the adjuvants and the equipotency ratio used in this study. Therefore, we aim to compare the efficacy and safety of propofol and thiopentone while inserting an LMA. Also, the price of thiopental is more feasible than propofol in India, which can lower the induction cost in daycare surgeries. It makes sense to utilize the less expensive medication if our investigation with IV thiopentone and IV propofol reveals equivalent or better insertion conditions for the LMA.

Hence, the present study is designed to compare the conditions to assist the insertion of the LMA with the commonly used agents thiopentone and propofol in their equipotent dosage after adequate initiation of midazolam and fentanyl. We hypothesize that IV thiopentone significantly facilitates the inserting conditions of the LMA when compared to IV propofol. Thus, the study aimed at assessing the effectiveness and efficacy of thiopentone compared to propofol for insertion of the LMA after adequate initiation of midazolam and fentanyl.

## Materials and methods

This is a prospective comparative study following a parallel randomized double-blinded technique with a 1:1 ratio carried out at the Anaesthesiology and Critical Care Department of Gauhati Medical College and Hospital (GMCH), Assam, from June 1, 2020, to May 31, 2021. The study included 80 American Society of Anesthesiologists (ASA) grade I and grade II patients with Mallampati scores (MPS) of 1 and 2 between 18 and 60 years of age of both sexes undergoing various elective minor surgeries (≤45 minutes) under general anaesthesia. The study approval was attained from the Institutional Ethical Committee of GMCH (No. MC/190/2007/Pt-11/Dec-2019/09). Each patient gave their informed written consent to participate in the trial.

Inclusion and exclusion criteria

The study included ASA grade I and grade II adult patients with MPS 1 and 2, aged 18-60 years of both sexes, undergoing a variety of elective minor surgeries (≤45 minutes) under general anaesthesia. Chronic smokers, hypertensive patients, patients with chronic obstructive pulmonary disease, bronchial asthma, diabetes, and drug allergy, patients with disorders of cardiovascular, hepatic, renal, and upper respiratory tract infections, anticipated difficult airways, and pregnant cases were excluded.

Eighty patients who met the criteria for inclusion were divided into two groups (A and B) by a computer-generated random selection using block randomization of size 40 each. Allocation concealment was ensured using thick sealed envelopes containing group A (propofol) or group B (thiopentone).

Methods

Study participants were put on fasting for at least six hours. Each patient received an alprazolam 0.25 mg tablet orally on the night before surgery. An 18G intravenous cannula was placed into a suitable vein in the forearm of the non-dominant hand after the patient was brought to the operating room. The area of the IV line was covered so that it was not visible to the anaesthetist collecting data. Ringer lactate (500 ml) was started at 8 ml/kg, and maintenance was done according to the Holliday-Segar formula. Standard monitoring such as non-invasive blood pressure (NIBP), electrocardiography (ECG), and pulse oximeter were attached. After this, the sealed envelope with patient allocation was opened. A junior resident not involved in any other aspect of the study prepared the study drugs as per the group allocation. The drugs were then handed over to the operation theatre (OT) technician. The baseline blood pressure levels, heart rate, and oxygen saturation were measured. Preoxygenation was started immediately with 100% oxygen with a face mask of appropriate size for the patient at 8 l/min for three minutes.

Pre-medication was started with an IV glycopyrrolate injection (0.2 mg) five minutes before the anaesthesia induction. Both groups received IV midazolam injection (0.04 mg/kg) followed by fentanyl injection (1.5 mcg/kg). Precisely one minute following injection, fentanyl was given, and the induction agent was administered by an associate anaesthetist who was not involved in the study. Propofol 2.5 mg/kg was administered to patients in group A, while thiopentone 5 mg/kg was administered to group B. Over 60 seconds, the induction agent was continuously injected. The efficacy of anaesthesia was evaluated after 30 seconds (loss of response to verbal commands and loss of eyelash reflex). If it was found to be satisfactory, the proper size classic LMA, according to the patient's weight, was selected for insertion using the standard technique [8]. The patients were in a supine position with their head and neck in a sniffing position. If the amount of anaesthesia was inadequate, repeat doses were administered as 0.25 mg/kg or 0.5 mg/kg, respectively, to a maximum dose of 3 mg/kg of propofol and 6 mg/kg of thiopentone [9,10]. Boluses required and time taken for successfully placing the LMA were noted. If insertion was not successful on the first attempt, a second attempt was taken. Total attempts required for successful insertion were recorded. LMA insertion was tried a maximum of two times during 90 seconds. If the second attempt failed, tracheal intubation using a muscle relaxant was performed, and the case was discarded.

Post-LMA classic insertion, the cuff was raised with a maximum optional air volume for that particular size. Ventilation was assisted in checking whether an adequate airway was secured and connected to the anaesthesia machine. Until three minutes after the insertion of the LMA, spontaneous ventilation was allowed, whilst anaesthesia was sustained with oxygen and nitrous oxide in a 1:2 ratio.

The primary outcome measures of the study were the required time for successful insertion, LMA insertion conditions, and overall response. The secondary outcome measures were pre and post-LMA insertion haemodynamic parameters, pulse rate, and NIBPs between the two groups.

The required time for successful insertion and the overall dose of an induction agent, including boluses, were recorded in seconds. The time needed for effective insertion was determined by timing the moment a square wave response on capnography appeared after the tip of the LMA bowl had passed past the lips.

The anaesthesiologist who inserted the LMA assessed the LMA insertion conditions [11] based on patient movement, laryngospasm, coughing, ease of insertion, and jaw opening [12]. Jaw opening was classified [13] as Grade 3 (full adequate jaw relaxation where LMA is done without difficulty), Grade 2 (partial inadequate jaw relaxation where LMA insertion requires bolus dose), and Grade 1 (no jaw relaxation where LMA insertion is not possible).

The ease of insertion was scored as Grade 3 (easy insertion at the initial attempt with no resistance), Grade 2 (some difficulty but successful at the second attempt), Grade 1 (impossible to insert), and LMA insertion unsuccessful [14]. Gagging, swallowing, and coughing were scored on a four-point scale [15], as Grade 4 (nil), Grade 3 (mild - if it was transient or minimal lasting < five seconds), Grade 2 (moderate - if it lasted more than five seconds but resolved within 20 seconds), and Grade 1 (severe - if it sustained or needed bolus dose to allow LMA insertion). Laryngospasm on insertion was graded as Grade 3 (nil), Grade 2 (partial, subsided by itself without any interventions), and Grade 1 (severe, requiring medication).

Head and limb movements were graded as Grade 4 (absent), Grade 3 (mild - some movement without affecting the positioning of LMA), Grade 2 (moderate - holding of LMA airway required, no need for any bolus doses), and Grade 1 (severe - additional doses of drug required).

The overall response was graded as per the modified scheme of Lund and Stovner: nil - no response; mild - gagging with or without coughing that settled within 30 seconds without intervention; moderate - limb and head movement, or any situation causing difficulty in LMA insertion that required an incremental dose); and severe - laryngospasm that required rescue drug succinylcholine [16].

At baseline, two minutes after administering midazolam and fentanyl (pre-LMA), and one, two, and three minutes after inserting the LMA, the haemodynamic parameters, pulse rate, and NIBPs (systolic, diastolic, and mean arterial pressure) were all recorded.

Statistical methods

The Statistical Package for the Social Sciences (SPSS) version 21 (IBM Corp., Armonk, NY) was utilized for data analysis considering p < 0.05 as significant. Categorical variables like insertion conditions and overall response between the two groups were evaluated using the chi-square test. In contrast, differences between continuous variables like age, weight, time taken for successful insertion, haemodynamic parameters, and blood pressures were tested using independent t-tests, paired and unpaired t-test, or their non-parametric counterparts, i.e., Mann-Whitney U test and Wilcoxon signed rank test, depending on the normality of the data. The data normality was assessed using the Kolmogorov-Smirnov test.

## Results

Out of the 80 patients, one was disqualified for monitor malfunction and one for operation theatre cancellation. At the same time, another patient refused to participate in the study. Thus, the study comprised 77 patients, including 39 in group A and 38 in group B.

Both groups had similar demographic characteristics with no statistically significant differences (p > 0.05). Also, no considerable difference was noted in height (p = 0.92), ASA grade (p = 0.70), and MPS scale (p = 0.93) between the groups (Table 1).

**Table 1:** Demographic characteristics of patients # presented as mean ± SD. Group A - propofol; Group B - thiopentone.

Variable	Group A	Group B	P-value
Sex (male:female)	15:24	17:21	0.93
Age (years)^#^	37.74 ± 12.69	35.44 ± 12.36	0.433
Weight (kg)^#^	55.17 ± 11.10	51.73 ± 9.23	0.14

The number of patients with complete jaw opening was higher in group A (32/39) than in group B (28/38). However, no statistically significant distinction was documented between the groups (p < 0.05). The LMA insertion was accessible in 89% of patients in group A. While in group B, 36.8% of patients presented difficulty in LMA insertion. The difference in ease of LMA insertion was significant between the groups. Only one patient in group B experienced mild coughing, whereas one in group A and four in group B experienced mild gagging. Laryngospasm was not observed in any of the patients. Mild movements were found in six and seven patients in group A and group B, respectively. Except for ease of insertion, other LMA insertion conditions showed no significant difference between the two groups (Table 2).

**Table 2:** LMA insertion conditions between the two groups LMA, laryngeal mask airway. Group A - propofol; Group B - thiopentone.

Conditions of LMA insertion	Grade	Description	Group A	Group B	P-value
Jaw opening	3	Full open	32	28	0.54
	2	Partial open	7	10	
	1	Nil	0	0	
Ease of insertion	3	Easy	34	24	0.029
	2	Difficult	5	14	
	1	Impossible	0	0	
Coughing	4	Nil	39	37	0.98
	3	Mild	0	1	
	2	Moderate	0	0	
	1	Severe	0	0	
Gagging/swallowing	4	Nil	38	34	0.33
	3	Mild	1	4	
	2	Moderate	0	0	
	1	Severe	0	0	
Laryngospasm	3	Nil	39	38	-
	2	Partial	0	0	
	1	Severe	0	0	
Partial movement	4	Absent	33	31	0.95
	3	Mild	6	7	
	2	Moderate	0	0	
	1	Severe	0	0	

As seen in Table 3, the mean time taken for LMA insertion was significantly higher in group B than in group A (p < 0.001).

**Table 3:** Time taken for LMA insertion LMA, laryngeal mask airway. Group A - propofol; Group B - thiopentone.

Parameter	Mean ± standard deviation		P-value
	Group A	Group B	
Time taken for LMA insertion (in seconds)	14.23 ± 3.11	16.15 ± 2.4	<0.001

In terms of overall conditions of LMA insertion, no statistically considerable difference was found between the groups (p > 0.05). In group A, no undesired responses occurred in 82% of patients compared to 68% in group B. Mild to moderate responses were encountered more in group B. Additional doses were required in six patients in group A and seven in group B to facilitate LMA insertion (Table 4). Out of 39 patients in group A, six patients (15.4%) required a second attempt for LMA insertion with additional doses. While in group B, seven out of 38 patients (18.4%) required a second attempt for successful LMA insertion.

**Table 4:** Overall responses to LMA insertion LMA, laryngeal mask airway. Group A - propofol; Group B - thiopentone.

Response grades	Group A (n = 39)	Group B (n = 38)	P-value
Nil	32	26	0.98
Mild	1	5	
Moderate	6	7	
Severe	0	0	

As seen in Table 5, the baseline and pre-medication (pre-LMA) heart rates in the groups were similar (p-value for t-test > 0.05). There was a decline in heart rate at the post-LMA at one minute, two minutes, and three minutes in both groups, although the reduction was noticeably greater in group A. The Student’s unpaired t-test showed that the decline in heart rate in group A was highly significant than in group B at one, two, and three minutes post-LMA (p < 0.001). Systolic, diastolic, and mean blood pressures were similar across the two groups, with a p-value of 0.05 or more at baseline and following pre-medication. However, after LMA, there was a decrease in systolic blood pressure (SBP), diastolic blood pressure (DBP), and mean blood pressure (MBP) in both groups, with the cases of group A experiencing a greater decline. The fall in SBP, DBP, and MBP at post-LMA at one minute, two minutes, and three minutes was statistically highly significant (p < 0.001).

**Table 5:** Sequential haemodynamic changes during LMA insertion # presented as mean ± standard deviation. LMA, laryngeal mask airway; SBP, systolic blood pressure; DBP, diastolic blood pressure; BP, blood pressure. Group A - propofol; Group B - thiopentone.

Variables	Group	Baseline	Pre-LMA	Post-LMA (1 min)	Post-LMA (2 min)	Post-LMA (3 min)
Heart rate	Group A^#^	82.66 ± 10.46	82.3 ± 8.06	74.17 ± 6.9	73.33 ± 5.93	71.87 ± 6.01
	Group B^#^	85.76 ± 8.09	85.52 ± 7.96	86.97 ± 6.80	85.09 ± 6.13	85.15 ± 7.42
	P-value	0.15	0.08	<0.001	<0.001	<0.001
SBP	Group A^#^	122.43 ± 9.68	116.8 ± 9.25	109.3 ± 7.41	100.12 ± 8.59	95.61 ± 9.47
	Group B^#^	122.52 ± 7.85	117.77 ± 8.7	117.07 ± 6.01	115.02 ± 7.28	111.98 ± 7.27
	P-value	0.96	0.63	<0.001	<0.001	<0.001
DBP	Group A^#^	80.15 ± 8.42	73.74 ± 8.22	69.58 ± 7.56	63.17 ± 7.62	61.35 ± 6.54
	Group B^#^	77.29 ± 7.12	75.02 ± 7.78	73.31 ± 7.31	71.96 ± 5.61	70.01 ± 6.02
	P-value	0.11	0.48	<0.001	<0.001	<0.001
Mean BP	Group A^#^	93.13 ± 8.19	88.94 ± 6.87	82.38 ± 8.24	75.7 ± 6.18	72.31 ± 5.8
	Group B^#^	91.84 ± 5.93	90.06 ± 6.28	88.84 ± 4.5	85.97 ± 5.14	84.02 ± 7.51
	P-value	0.43	0.46	<0.001	<0.001	<0.001

## Discussion

Endotracheal intubation is a routine procedure to conduct general anaesthesia and a secure way of controlling the airway. Still, laryngoscopy and tracheal intubation produce a stress response that leads to a reflex surge in sympathoadrenal activity. This results in dysrhythmia and a rise in heart rate and blood pressure, which are lethal to cardiac patients. Face masks are habitually used for short surgical procedures during induction and maintenance under total intravenous anaesthesia and volatile induction. But it has the shortcoming of holding the mask continuously in spontaneously breathing patients.

LMA started gaining popularity as a substitute for endotracheal intubation and facemask because it causes less haemodynamic variations, is associated with a negligible rise in intraocular pressure, decreases the likelihood of sore throat, and frees the anaesthesiologist's hands to perform other important tasks during the surgical procedures. In developing countries, daycare surgeries significantly reduce costs [17]. Lesser complications and airway morbidity were reported with LMA making early discharges and shorter hospital stays [18]. Attempts have been made in the current study to compare and evaluate the suitability of conditions for the insertion of the LMA using the drug as specified in two groups after pre-induction doses of IV midazolam and IV fentanyl.

The two groups were comparable in age, gender, weight, ASA physical status, and MPS. This finding was similar to various other studies [5,14,19].

The total number of patients with full jaw opening in the current study was higher in group A than in group B. However, the disparity was insignificant (p < 0.05). The results align with a different study that compared the conditions surrounding the LMA insertion in 70 unmedicated patients treated with either midazolam-alfentanil-thiopentone or midazolam-alfentanil-propofol [20]. Although full jaw opening was not found statistically significant, the clinical significance of the finding is essential [21].

Also, we observed that the ease of LMA insertion was significantly different between the groups. The LMA insertion was comparatively more effortless in group A than in group B (p < 0.05). Numerous other investigations reported similar findings [11,20].

Coughing, gagging, and patient movements were observed more among our study patients in group B. Similar conditions were experienced in another study [4]. A study comparing midazolam-alfentanil-thiopentone and midazolam-alfentanil-propofol for LMA insertion observed more coughing, gagging, or laryngospasm in group B. But these interpretations were not statistically significant, which agrees with other findings [20]. Laryngospasm was absent in our study. In a study by Vandana et al., 12% of patients in group B had laryngospasm and airway blockage, but none in group A. Another study comparing group A and group B with lignocaine spray for easy insertion of the LMA also agrees with our results [21].

The mean (±SD) time taken for LMA insertion in patients of group B was 16.15 (±2.4) seconds, which was notably higher (p < 0.001) than in patients of group A. A study comparing patients like that of group A vs. group B in daycare surgery found the meantime (in seconds) in group A as 16.6 (±11.6) and 18.2 ± 12.8 seconds in group B; however, the variation was not significant statistically [22].

Further, in comparing the smooth insertion of the LMA with group A and group B when paired with midazolam, it was discovered that 93.3% of patients of group A had successful LMA insertion on the first attempt, compared to 84.4% in group B [23]. These observations were almost comparable to our study.

Talwar et al. evaluated the haemodynamic alterations in individuals similar to the present study's group A or group B during LMA insertion and immediately afterwards. They observed a drop in the heart rates and arterial blood pressure following insertion in both groups, with patients in group A recording a higher drop than group B, which is consistent with our findings [11]. Another study reported that despite the two groups' baseline heart rates being comparable, there was a drop in post-LMA heart rates and arterial blood pressures (systolic, diastolic, and mean) at one minute, two minutes, and three minutes in both groups. This shrinkage was more pronounced in group A than in group B. These very significant (p < 0.0001) findings support the conclusions of our investigation [24].

Limitation

The study was a single hospital-based study. Also, patients with various co-morbidities were barred from the study. Multicentre studies with a larger sample size may help gain a broad view of the conclusion of the present study.

## Conclusions

LMA insertion was significantly easy with patients in group A receiving propofol. The induction also requires less time as compared to group B patients receiving thiopentone. However, patients in group A had a fall in their haemodynamic parameters compared to group B.

The combination of midazolam, fentanyl, and propofol appears to be marginally superior to the combination of midazolam, fentanyl, and thiopentone to facilitate insertion of the LMA due to its improved ease of insertion, the shorter time required for insertion, and better recovery profiles.
